# Miscellaneous Items

**Published:** 1867-11

**Authors:** 


### *A Quack in an "Abject Condition."*

In London a quack doctor has been in the habit of sending indecent pamphlets to respectable people. In one instance, however, he got his deserts, as shown by this communication in the *Pall Mall Gazette:* "Sir—I have been frequently annoyed by receiving Dr. Jordan's productions. Last night, during dinner, one arrived. Thinking that it was a tradesman's advertisement, I was on the point of giving it to a young lady who was sitting next to me, when the name of 'Jordan' caught my eye. This morning I paid the Doctor a visit, at 29 George Street, Hanover Square. I remained with him for a few minutes, and left him apparently suffering from "nervous exhaustion." I recommend other men who are annoyed by his abominations to pay him a visit after the receipt of the next pamphlet, and leave him in the same abject condition."

Liebig's Artificial Milk is in imitation, as close as chemistry can make, of the natural food of the human infant. It is prepared as follows: Half an ounce of wheat flour is boiled to a paste in five ounces of skimmed milk. To this is added immediately a mixture of one-half ounce of bruised malt, one ounce of water and three grammes of a solution of two parts of bi-carbonate of potassa in eleven parts of water. The whole is then kept warm by standing within an an envelope of tepid water until it is no longer pastry, but of a creamy consistence. After fifteen or twenty minutes it is put on a fire for a few seconds only, and then strained through a hair sieve. It should be allowed to stand long enough to deposit some fibrous matter before it is given to the child or invalid.—*Phil. Med. Reporter.*

*Heat as a Resuscitation Agent.*—Dr. J. G. Richardson, in the *Am. Jour.*, urges from his observation and experiments the importance of direct Heat as an agent in the restoration of still-born infants, in cases of asphyxia from drowning, hanging, or the inhalation of noxious gases, especially the vapor of Chloroform. He advises not merely *warming* the surface but artificially warming the blood within the limbs of the persons apparently dead, and then propelling by frictions toward the heart as rapidly as possible. The heart's pulsations in many instances really take place, feebly, for a considerable time ofter being undiscoverable by external observation. The heat shoult approximate *roasting* as nearly as may be without positive destruction of the tissues. Mere vesication he thinks ought not to be considered more than a minor evil in comparison with the cessation of life.—*Chicago Med. Examiner.*

### *Extraordinary Fecundity.*

Dr. Becker-Lawrich de Ronneburg communicates to the Society of "Gynecologie," under the title of extraordinary fecundity, the history of a woman married twelve years, and now pregnant for the nineteenth time. At the outset, she was confined at the full term; then she aborted nine times in succession at four months * * * was delivered subsequently at eight months; and after that aborted anew seven times in succession at the fourth month. At the present time she is again pregnant, but alleges that she feels the precursory symptoms of abortion, such as a sero-sanguinolent discharge, &c. With her numerous and fatiguing occupations, it is not probable that she will now go the full term. Nevertheless, in spite of the copious hæmorrhage which accompanies her miscarriages, and which have often endangered her life, she is fat and well nourished.—*L' Union Medicale.*

The prevalence of cholera among the troops on Governor's Island, New York Harbor, during the past summer, and at the West, in some localities where it prevailed last year, tends to confirm the opinion that the seeds of the disease may survive the winter in warm or temperate regions, unless disinfecting agents are employed most thoroughly for their extinction.

M. Nelaton has resigned his chair as Professor of Clinical Surgery. The reason assigned is, that, in addition to his previously immense practice, the recent death of Jopert de Lamballe and Velpeau, has put upon him a large amount of work. We have heard M. Nèlaton's professional income, from private practice, his positions as surgeon to the Emperor and professor agrègé, estimated at $126,000 per annum. It is said that the anatomist, Sappéy, will succeed him in the clinical chair.—*Medical Gazette.*

Another prominent physician of Paris is dead, Dr. Bouley. He was not known as a writer but had considerable local celebrity for his attainments. The mortality among foreign scientific men during the year is remarkable. In London, Lawrence, Faraday and Bazire; in Paris, Jobert de Lamballe, Trousseau, Rayer, Velpeau Gibert, Follin, Chartroule and now Bouley.—*Ibid.*

*Copper for Cholera.*

Dr. V. Burq having opserved in 1852 that about 200 persons working in and around a copper foundry were not attacked by cholera, even during the worst stages of the epidemic, made further inquiries, and found that all persons handling this metal, whom he met, enjoyed the same immunity. He therefore concluded to try the use of copper as a medicine for those attacked by cholera. He administered sulphate of copper internally, and applied metallic copper externally. It was asserted by some medical authorities that the plan was not successful, but a late communication to the French Academy, by Dr. Lisle, of the lunatic asylum at Marseilles, contains the statement that he had cured 20 out of 24 patients by administering sulphate of copper, even in smaller doses than those prescribed by Dr. Burq.

The Italian government has been the first to recognize by law the devotion and disinterestedness of the medical profession in their attendance upon cases of cholera. By a recent law the Chambers have provided that an annual pension of four hundred francs shall be paid to the widow, and a thousand francs to the children of any physician who dies from such exposure. The pension of the widow ceases if she marries again, and that of the children on attaining majority. —*Boston Med. and Surg. Jour.*

A New York religious newspaper says that the late Dr. James Jackson, of Boston, was "emphatically the poet and philosopher of the medical profession!"

Dr. J. Marion Sims has had conferred upon him by the King of Italy the Order of St. Maurice and Lazarus, and by the Queen of Spain he has been named Commander, of the first class, of the Order of Isabel la Catholique, as recognitions of the value of his late medical work.—*Boston Med. and Surg. Jour.*

*Population of France.*—At the close of the discussion in the French Academy, on the growth of the population, it seems conceded that while the population is increasing sensibly, the rate of population is gradually diminishing. In comparing the fact with the growth of population in neighboring countries, M. Guerin concludes that it involves a serious danger for France, calling for a remedy.—*Pacific Med. and Surg. Jour.*

*Caviale's Collection of Calculi.*—Not long before his death, Caviale exhibited to the French Academy, his collection of urinary calculi, from 2700 patients operated on by him during the 43 years of his professional career. In 1600 of the number he had performed his favorite operation of Lithotrity.—*Pacific Med. and Surg. Jour.*

*Croup Treated by Sulphur.*—M. Lagauterie (*Half Yearly Abstract*) gives in Croup, teaspoonful doses, every hour, of a mixture of sulphur and water (a teaspoonful to a glass of water) with effects which he describes as wonderful. The cure, in seven very severe cases, was accomplished in two days, the only symptom remaining being a slight cough.—An observation of the effect of sulphur on the oidium of vines, led to its use in croup.—*Pacific Med. and Surg. Jour.*

*Lactate of Zinc in Epilepsy.*—Dr. Hart (*Chicago Med. Jour.*) has tried this remedy in combination with belladonna, on 240 patients in the Western Lunatic Asylum of Kentucky, all of whom had been affected with epilepsy from three to six years. An improvement took place in all, and in no case did he use it "without effectually controlling the paroxysm in from 24 to 48 hours." His formula was: ℞ Zinci lactatis, gr. xxx.; Ext. Belladonnæ, gr. viii. M. ft. pil. x. Sig. One before each meal.

